# Suppression of Inflammatory Mediators by Cruciferous Vegetable-Derived Indole-3-Carbinol and Phenylethyl Isothiocyanate in Lipopolysaccharide-Activated Macrophages

**DOI:** 10.1155/2010/293642

**Published:** 2010-04-13

**Authors:** Jo-Ting Tsai, Hui-Ching Liu, Yue-Hwa Chen

**Affiliations:** ^1^Department of Radiation Oncology, Taipei Municipal Wan-Fang Hospital, Taipei Medical University, Taipei 116, Taiwan; ^2^School of Nutrition and Health Sciences, Taipei Medical University, 250 Wu-Hsing Street, Taipei 110, Taiwan

## Abstract

This study was aimed to examine the effects of indole-3-carbinol (I3C) and *β*-phenylethyl isothiocyanate (PEITC), bioactive components present in cruciferous vegetable, on the production of inflammatory mediators, including nitric oxide (NO), tumor necrosis factor-*α* (TNF-*α*) and interleukin-10 (IL-10), in lipopolysaccharide- (LPS-) stimulated RAW 264.7 macrophages. Possible mechanisms of the NO-inhibitory effects were also explored. The results indicated that I3C and PEITC inhibited NO production, and this suppression was associated with decreased production of TNF-*α* and IL-10 by activated macrophages. In addition, I3C suppressed NO production even after the inducible nitric oxide synthase (iNOS) protein had been produced, but such an inhibitory effect was not observed in cells treated with PEITC. Furthermore, both compounds reduced the NO contents generated from an NO donor in a cell-free condition, suggesting that the increased NO clearance may have contributed to the NO-inhibitory effects. In summary, both I3C and PEITC possessed antiinflammatory effects by inhibiting the productions of NO, TNF-*α*, and IL-10, although the NO-inhibitory effects may have involved in different mechanisms.

## 1. Introduction

Macrophages play important roles in a host's immune defense system during infection as well as in the processes of disease development. Activation of macrophages by stimuli, such as the bacterial endotoxin, lipopolysaccharide (LPS), and viruses, increases the production of numerous inflammatory mediators, including nitric oxide (NO), prostaglandin E_2_ (PGE_2_), and various cytokines [1]. NO is a small radical molecule that possesses many physiological functions, being involved in vasorelaxation, neurotransmission, immunoregulation, and inflammation. NO is synthesized from the amino acid L-arginine by nitric oxide synthase (NOS) in various cells and tissues. Among isoforms identified, the inducible form of NOS (iNOS) is mainly expressed in macrophages and lymphocytes, and is synthesized by stimulants such as bacterial endotoxin lipopolysaccharide (LPS) and cytokines, including interferon-*γ* (IFN-*γ*), interleukin-1 (IL-1), and tumor necrosis factor-*α* (TNF-*α*) [2]. Low concentrations of NO produced by iNOS are involved in antipathogenic and antitumor responses of macrophages. However, prolonged overproduction of NO and the dysregulated expressions of cytokines during chronic inflammation have been implicated in several pathological conditions, such as autoimmune diseases and cancers [2–4]. Hence, agents that regulate the production of cytokines and suppress the overproduction of NO may possess protective roles in inflammation-related diseases.

There is increasing interest in using natural products to modulate immune responses and neutralize inflammatory processes because of their fewer side effects and lower cytotoxicities [5, 6]. Cruciferous vegetables, including broccoli, cauliflower, cabbage, and mustard, are reported to play protective roles in different diseases. The high contents of glucosinolates and glucosinolate derivatives, including isothiocyanates and indoles, were implicated as being responsible for the biological effects of these vegetables [7–9]. Both isothiocyanate and indole derivatives, including phenylethyl isothiocyanate (PEITC), sulforaphane, and indole-3-carbinol (I3C), are reported to suppress NO production by inhibiting inducible nitric oxide synthase (iNOS) expression in activated RAW 264.7 macrophages [10–12]. Although sulforaphane and PEITC were shown to decrease TNF-*α* production and interleukin-1*β* (IL-1*β*) mRNA expression, respectively, in LPS-activated macrophages [10, 11], there is little information on the effects of I3C on cytokine production. Besides, the effects of PEITC or I3C on these inflammatory mediators are usually examined individually, not simultaneously, or compared under the same conditions. Because the productions of NO and cytokines are closely associated, this study attempted to examine and compare the effects of PEITC and I3C on the productions of NO and cytokines, including the proinflammatory TNF-*α*, and the antiinflammatory IL-10, in LPS-activated RAW 264.7 cells. To obtain further insights into the NO-suppressive effects of these compounds, indirect NOS enzyme activities and in vitro NO clearance activities were also investigated. Results from this study may increase our understanding of how compounds from cruciferous vegetable modulate immune functions, and the potential use of these compounds in inflammation-related diseases.

## 2. Materials and Methods

### 2.1. Chemicals and Biochemicals

I3C, PEITC, LPS, sulfanilamide, N-(1-naphthyl)ethylenediamine dihydrochloride, and sodium nitrite were commercially obtained from Sigma Chemical (St. Louis, MO). Absolute ethanol was purchased from Merck (Darmstadt, Germany). Dulbecco's modified Eagle medium (DMEM) and fetal bovine serum (FBS) were from GIBCO (Grand Island, NY). All other laboratory chemicals were purchased from Sigma and USB (Cleveland, OH).

### 2.2. Cell Culture

Murine monocyte-macrophage RAW 264.7 cells were obtained from the Biosource Collection and Research Center, Hsinchu, Taiwan (BCRC 60001). Cells were grown as monolayers in DMEM supplemented with 10% FBS at 37°C in an atmosphere of 95% air and 5% CO_2_. I3C and PEITC were dissolved in absolute ethanol, and the concentration of absolute ethanol added to the media never exceeded 0.01% (v/v). 

### 2.3. Assays for NO and Cytokine Production and Release

Nitrite concentration in the culture medium was quantified spectrophotometrically after its reaction with the Griess reagent (a 1 : 1 mixture of 1% sulfanilamide/5% H_3_PO_4_ and 0.1% naphthylethylenediamine dihydrochloride) [13]. The production and release of cytokines in the culture medium were determined using commercial enzyme-linked immunosorbent assay (ELISA) kits (Amersham Biosciences, Buckinghamshire, UK) following the manufacturers' instructions.

### 2.4. In Vitro NO Clearance Activity

NO clearance activities of these compounds were determined according to a method modified from by Marcocci et al. [14]. Basically, I3C and PEITC were incubated in an NO donor solution of sodium nitroprusside (SNP) (10 mM) at room temperature for 30 minutes in a cell-free condition, and the nitrite concentration in the reaction mixture was determined using the Griess reagent.

### 2.5. Statistical Analysis

Values are expressed as the mean ± standard deviation (SD), and were analyzed using SAS software versus 6.12 (SAS Institute, Cary, NC). One-way analysis of variance (ANOVA) followed by Fisher's least significant difference test and Student's *t*-test was used to determine statistical differences between groups. The significance of the mean differences was based on a *P* value of <.05.

## 3. Results and Discussion

### 3.1. Effects of I3C and PEITC on LPS-Stimulated NO Production

**Figure 1:**
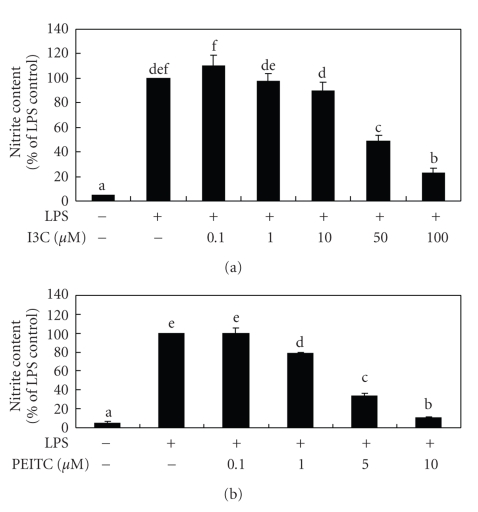
Effects of I3C (a) and PEITC (b) on lipopolysaccharide- (LPS-) induced NO released into the medium by RAW 264.7 cells. Cells were treated with LPS (330 ng/mL) and various concentrations of I3C and PEITC for 24 hours. The nitrite contents in the medium were then determined using the Griess reagent. Values represent the mean ± SD from three measurements, and LPS group is regarded as 100%. Data for the same parameter with different letters (a–f) significantly differ (*P *< .05).

Figure 1 shows that I3C and PEITC inhibited 52% to 77% and 22% to 89% of LPS-stimulated NO production, respectively. Because no cytotoxic effect was observed after cells were treated with LPS in the presence of I3C or PEITC for 24 hours, the NO-inhibitory effects by I3C and PEITC were not due to cytotoxicity. Comparing to the results obtained from macrophages that were activated by LPS/ IFN-*γ* [12], greater NO-inhibitory potencies were observed in LPS-activated macrophages, indicating that IFN-*γ* might generate more complicated responses on NO production in macrophages. 

**Figure 2:**
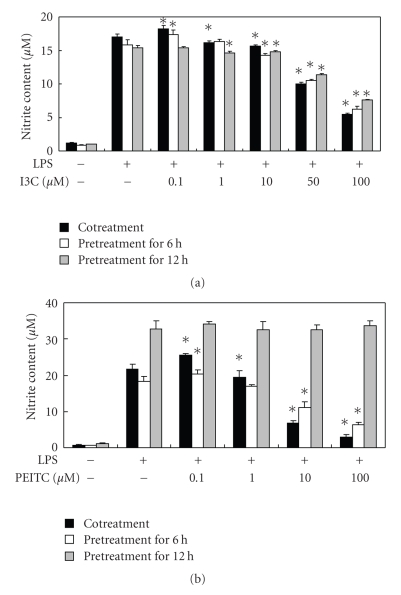
Effects of I3C (a) and PEITC (b) on NO production in lipopolysaccharide- (LPS-) pretreated with RAW 264.7 cells. Cells were pretreated with LPS (330 ng/mL) for 0, 6, or 12 hours, followed by the addition of various concentrations of I3C and PEITC for up to 24 hours. The nitrite content in the medium was then determined by the Griess reagent. Values represent the mean ± SD from three measurements. An asterisk (*) indicates a significant difference from the corresponding LPS control group (*P *< .05).

**Figure 3:**
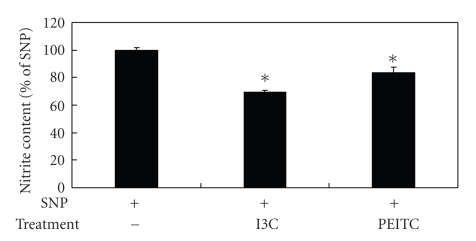
Effects of I3C and PEITC on in vitro NO clearance activity. I3C (50 *μ*M) and PEITC (5 *μ*M) were incubated in a 10 mM sodium nitroprusside (SNP) solution for 30 minutes, and the nitrite content in the reaction mixture was then determined using the Griess reagent. Values represent the mean ± SD from three measurements. An asterisk (*) indicates a significant difference from the control group (*P *< .05).

Although evidence demonstrated that the decreased NO production by I3C and PEITC is mediated through the suppressing iNOS protein expression in activated macrophages [12, 15], other possibilities may exist. Because the expression of iNOS may be also regulated at the posttranslation level [16], iNOS enzyme activity was indirectly evaluated. After being activated with LPS for different periods of time, macrophages were treated with I3C or PEITC for up to 24 hours, and the NO content in the culture medium was examined. The results showed that LPS-stimulated NO production was inhibited by I3C treatment regardless of the pre- or cotreatment with LPS, although cotreated cells exhibited a greater extent of inhibition (Figure 2(a)), suggesting that I3C interfered with iNOS's enzyme activity. PEITC, on the other hand, showed a distinct pattern in LPS-treated cells, where no inhibition was noted in 12-hour LPS-pretreated cells, less inhibition was observed in 6-hour pretreated cells, and the maximal NO suppression occurred in cotreated cells (Figure 2(b)), indicating that PEITC had no impact on iNOS enzyme activity once the protein was synthesized. NO is a small molecule with free radical characteristics, so it readily interacts with other compounds. Because I3C was reported to be capable of acting as a scavenger of free radicals [17], in vitro NO clearance activity of these compounds was also examined. As shown in Figure 3, both I3C (50 *μ*M) and PEITC (5 *μ*M) decreased the levels of NO generated from the NO donor. Therefore, I3C and PEITC might directly interact with NO, thus decreasing its availability. Blomhoff [18] suggested that cruciferous vegetables are dietary plants rich in compounds that can reduce oxidative stress, including reactive nitrogen species (RNS), which may be formed from excess NO free radicals, and I3C was shown to serve as a scavenger of free radicals and inhibit lipid peroxidation [17, 19]. Furthermore, glutathione S-transferase (GST), a phase II xenobiotic metabolism enzyme which can be induced by I3C or PEITC, was indicated to be able to inactivate NO [20], so I3C and PEITC may also indirectly eliminate NO through the induction of GST. Alternatively, LPS-induced macrophage activation was reported to require the LPS receptor complex, which plays essential roles in binding and in mediating the response to LPS [21]. Therefore, binding interference of I3C or PEITC with either LPS and/or the LPS receptor complex cannot be ruled out. Nevertheless, PEITC did not show any impact on NO production when macrophages were pretreated with LPS for 12 hours (Figure 2(b)). A higher NO concentration generated from the LPS-treated cells (18.31 ± 0.60 *μ*M) comparing to the in vitro condition (8.96 ± 0.08 *μ*M) may explain the discrepancy, because the NO concentration was too high for PEITC to react without inhibited iNOS activity. On the other hand, the binding of intracellular proteins by PEITC [22] may also decrease its bioavailability on NO clearance. Taken together, different mechanisms may be involved in the NO-inhibitory effects of I3C and PEITC. I3C may act through decreasing iNOS expression, interfering with iNOS enzyme activity, and directly trapping NO to decrease NO availability, whereas PEITC mainly acts through decreasing iNOS expression and directly interacting with NO. Nevertheless, the molecular mechanisms of NO's inhibitory effects against these derivatives require further investigation.

### 3.2. Effects of I3C and PEITC on the Production of Cytokines

**Figure 4:**
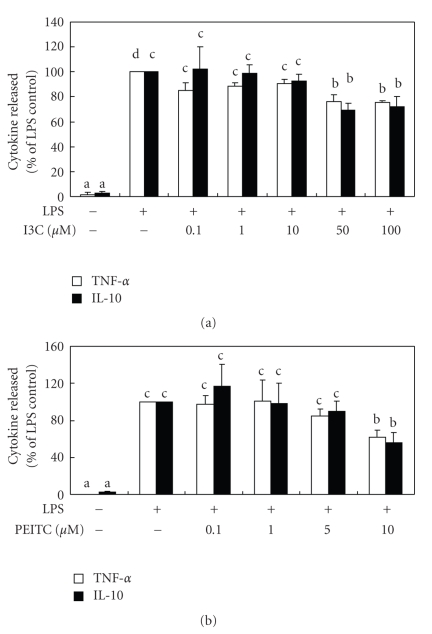
Effects of I3C (a) and PEITC (b) on lipopolysaccharide- (LPS-) activated cytokines released into the medium by RAW 264.7 cells. Cells were cotreated with LPS (330 ng/mL) and various concentrations of I3C and PEITC for 24 hours. The levels of tumor necrosis factor-*α* (TNF-*α*) and interleukin-10 (IL-10) in the medium were then determined using commercial ELISA kits. Values represent the mean ± SD from three measurements. Data in the same cytokine group with different letters significantly differ (*P *< .05).

The production of NO can be regulated by various cytokines, so the release of the proinflammatory TNF-*α* and the antiinflammatory cytokine, IL-10, was determined. As shown in Figure 4, LPS significantly enhanced the productions of TNF-*α* and IL-10 by RAW 264.7 cells, and high concentrations of I3C and PEITC suppressed such enhancements of both TNF-*α* and IL-10. The expression of iNOS can be induced by proinflammatory cytokines, such as TNF-*α* [23], and inhibited by antiinflammatory cytokines, including IL-10 [24]. It is reasonable to assume that the NO-inhibitory effects of these compounds may occur through suppressed production of TNF-*α* or through increased production of IL-10. Nonetheless, both TNF-*α* and IL-10 were significantly inhibited by high concentrations of I3C and PEITC in LPS-stimulated macrophages, suggesting that IL-10 might not play a crucial role in I3C- and PEITC-induced NO inhibition. Because NO production may be regulated by a variety of mechanisms as discussed, whereas TNF-*α* and IL-10 inhibition may rely on suppression of gene expression, that is, via NF-*κ*B signaling pathway, the inhibitory effects on TNF-*α* and IL-10 production by I3C and PEITC are not as potent as that of NO production. On the other hand, a variety of cytokines can be released after treatment with LPS, and are mutually regulated; hence other cytokines, in addition to TNF-*α* and IL-10, may act additively or synergistically to affect NO production. Furthermore, cytokines not only modulate NO production, but NO may also modulate the secretion of cytokines. NO was shown to modulate the production of IL-2 and IFN-*γ* by T cells [23] as well as TNF-*α* and IL-1*β* in activated alveolar macrophages [25], and this may partially explain the effects of I3C and PEITC on the production of cytokines. Finally, different studies indicated that the expression of IL-10 in cultured macrophage might not reflect what is observed in animals [26, 27]. For example, an immunomodulator, SR31747A, downregulates both NO and IL-10 expressions in LPS-activated RAW 264.7 macrophages, but it enhances IL-10 production in a murine model of sepsis [27]. Thus, the results obtained from this study are informative, but require further investigation.

In conclusion, I3C and PEITC possess antiinflammatory effects by inhibiting the productions of NO, TNF-*α*, and IL-10 in LPS-stimulated macrophages. NO's inhibitory effect on I3C, in addition to decreasing the expression of iNOS, may at least partly be through interfering with iNOS enzyme activity as well as direct NO clearance activity, whereas PEITC may act through its direct NO clearance activity but not interfere with iNOS enzyme activity.
